# Gremlin is Overexpressed in Lung Adenocarcinoma and Increases Cell Growth and Proliferation in Normal Lung Cells

**DOI:** 10.1371/journal.pone.0042264

**Published:** 2012-08-01

**Authors:** Michael S. Mulvihill, Yong-Won Kwon, Sharon Lee, Li Tai Fang, Helen Choi, Roshni Ray, Hio Chung Kang, Jian-Hua Mao, David Jablons, Il-Jin Kim

**Affiliations:** 1 Thoracic Oncology Laboratory, Department of Surgery, University of California San Francisco, San Francisco, California, United States of America; 2 Comprehensive Cancer Center, University of California San Francisco, San Francisco, California, United States of America; 3 Life Sciences Division, Lawrence Berkeley National Laboratory (LBNL), Berkeley, California, United States of America; Univ of Bradford, United Kingdom

## Abstract

**Background:**

Gremlin, a member of the Dan family of BMP antagonists, is a glycosylated extracellular protein. Previously Gremlin has been shown to play a role in dorsal-ventral patterning, in tissue remodeling, and recently in angiogenesis. Evidence has previously been presented showing both over- and under-expression of Gremlin in different tumor tissues. Here, we sought to quantify expression of Gremlin in cancers of the lung and performed in vitro experiments to check whether Gremlin promotes cell growth and proliferation.

**Methodology/Principal Findings:**

Expression of Gremlin in 161 matched tumor and normal lung cancer specimens is quantified by quantitative real-time PCR and protein level is measured by immunohistochemistry. *GREM1* was transfected into lung fibroblast and epithelial cell lines to assess the impact of overexpression of Gremlin in vitro.

**Results:**

Lung adenocarcinoma but not squamous cell carcinoma shows a significant increase in Gremlin expression by mRNA and protein level. Lung fibroblast and epithelial cell lines transfected with *GREM1* show significantly increased cell proliferation.

**Conclusions/Significance:**

Our data suggest that Gremlin acts in an oncogenic manner in lung adenocarcinoma and could hold promise as a new diagnostic marker or potential therapeutic target in lung AD or general thoracic malignancies.

## Introduction

The discovery of molecular alterations specific to cancerous and pre-cancerous cells has yielded insight into the role played by oncogenes and tumor suppressor genes in the initiation and progression of human cancers [1], [2]. Frequently, oncogenes are derived from proto-oncogenes in processes such as point mutations, gene amplifications, or gene rearrangements [3], [4]. These structural changes leading to the development of an oncogene then result in quantitative and qualitative changes in the expression of the related protein product.

In lung cancer, important oncogenes have previously been identified and used for targeted therapy. *EGFR* is mutated in around 20% of lung adenocarcinoma (AD) patients [5]. Patients with *EGFR* mutations have shown a positive response to therapy with erlotinib, although many of these patients relapse later, frequently due to a secondary *EGFR* mutation, T790M [6]. An oncogenic fusion gene, *EML4-ALK*, was recently identified [7]. Crizotinib can now be used for the treatment of patients with the *EML4-ALK* fusion [8]. However, the frequency of *EML4-ALK* in the Western population is only around 1–7% [7], which means that more than 40% of non-small cell lung cancer (NSCLC) patients without *EGFR* or *EML4-ALK* mutations are left without any available targeted therapy [7]. As such, there is an urgent need for the development of new diagnostic markers and potential therapeutic targets to reduce the mortality of lung cancer.

To identify novel genes that may potentially play a role in carcinogenesis, we sought to identify genes that were highly upregulated in comparison to matched normal tissue. Gremlin (*GREM1*) was one of the best candidates with significant overexpression in lung cancer compared to matched normal tissues in several published adenocarcinoma microarray datasets [9], [10]

Gremlin was initially identified in a *Xenopus* expression cloning screen and referred to as *drm* (down-regulated in *mos-*transformed cells) [11], [12]. A 25 kDa protein, it carries a carboxy-terminal cysteine-rich motif that is homologous with a protein domain shared by members of secreted proteins such as DAN and Cerberus [13]. *Drm* was identified as a novel gene that is suppressed in cells transformed by v-ras, v-src, v-raf, and v-fos. It was shown that DRM can inhibit the growth of normal but not transformed cells in culture [11]. A possible tumor-suppressor role was proposed for *drm* on the basis of its down-regulation in these transformed cell lines and it was hypothesized that high levels of *drm* inhibit the growth or viability of normal cells, but that transformed cells are resistant to this inhibitory effect [11].

Studies of normal development of the limb have implicated a role for Gremlin in proper establishment of limb bud morphology. Gremlin indirectly enhances FGF-mediated limb outgrowth while simultaneously inhibiting chondrogenesis and cell death [14], [15]. Sonic Hedgehog (SHH) has also been shown to upregulate and maintain Gremlin expression, enabling Gremlin to relieve the repressive effects of BMP-4 on FGF-4 expression, yielding a net positive feedback to increase SHH [14], [16].

The importance of the interaction between Gremlin and the BMP signaling pathway in the normal development of the proximal-distal patterning of the lung has previously been investigated. Gremlin acts as a functional physiological antagonist that restricts BMP-4 activity to the distal bud, thereby regulating the number of branching epithelial sacs [17]. Similarly, antagonism of BMP-4 signal using the BMP antagonist *Xnoggin* results in a severe reduction in distal epithelial cell types and an increase in proximal cell types [18]. Overexpression of Gremlin in the distal lung epithelium using an SP-C promoter in mice results in transgenic lungs that phenotypically resemble proximal airways epithelium with decreased squamous epithelium [19]. Deletion of *GREM1* in mouse embryonic stem cells results in a neonatal lethal phenotype characterized by a reduction in differentiated alveoli and multi-layered epithelium in comparison to wild-type embryos [2].

RNA and protein analysis indicates that Gremlin is frequently undetectable in multiple malignant cell lines, including neuroblastoma, glioblastoma, medulloblastoma, and colon adenocarcinoma [20]. The pattern of expression suggests a possible role as an inhibitor of tumor progression in these lineages. Overexpression of Gremlin was sufficient to inhibit the neoplastic phenotype of both Daoy and Soas-2 cell line [21]. Analysis of publicly-available microarray data also suggests a significant downregulation of Gremlin in tumors of the CNS [22].

While these studies have suggested a possible tumor-suppressive role for Gremlin, recent work has shown an oncogenic role for Gremlin in other tumor types. Gremlin is over-expressed in stromal cells associated with basal cell carcinomas of the skin, and can promote cell proliferation in this model [23]. Gremlin is also upregulated in the lung cancer cell line A549 per a semi-quantitative northern blot analysis [24]. It was recently reported that Gremlin is overexpressed in many malignant mesothelioma (MM) tissue specimens [25]. Inhibition of Gremlin via shRNA significantly inhibited proliferation of MM cell lines.

The function of Gremlin in cancer cells is controversial and appears to work in a tissue-specific manner. In lung cancer, nothing to date is known about the role of Gremlin. Thus, we performed genetic and histological analysis of Gremlin in a large number of lung AD and squamous cell carcinoma (SCC) samples. mRNA and protein expression analysis of Gremlin were done in 161 matched tumor-normal pairs. Of these, 96 pairs were of AD, while 65 of the pairs were from SCC. The potential oncogenic function of Gremlin was analyzed by *GREM1* transfection in multiple normal lung fibroblast and epithelial cells.

## Results

### Expression Analysis of Gremlin in Published Microarray Data

**Figure 1:**
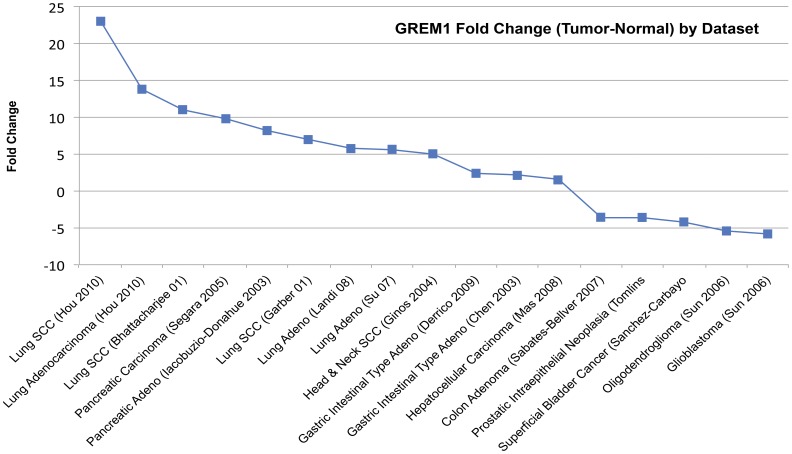
Fold-change in Gremlin expression between tumor and normal sample by microarray dataset. Datasets containing tumor and normal sample and probes for GREM1 were selected and a two-tailed, unpaired t-test was conducted between log-transformed and median-centered expression values for GREM1 probes in tumor and normal samples. Datasets representing 17 tumor-normal comparisons contained significant (p<.001) differences in GREM1 expression between tumor and normal samples.

A systematic review of publicly available microarray data within the Oncomine database (www.oncomine.org) was conducted. Appropriate datasets were identified using a search for tumor – normal comparisons of datasets containing probes targeting *GREM1* (Gremlin). Expression values were log-transformed and median-centered per array. Our search identified 24 datasets appropriate for analysis. Comparison of fold-change (t-test, p<.001) between tumor and normal yielded 4 lung cancer datasets in which Gremlin expression was significantly changed. Two datasets involving lung adenocarcinoma specimens with matched normal tissue showed significant upregulation [9], [19], as did two squamous cell carcinoma datasets that did not utilize paired tumor – normal specimens, instead providing normal specimens matched to adenocarcinoma samples [26], [27]. A total of 17 datasets with significant fold-change in Gremlin expression between tumor and normal were identified. Cited literature was then reviewed to confirm that the analysis was as documented in the Oncomine annotation. Fold-change representation of change in Gremlin expression across datasets available in Oncomine is included in figure 1.

### Gremlin is Overexpressed in Lung AD, not SCC

**Figure 2:**
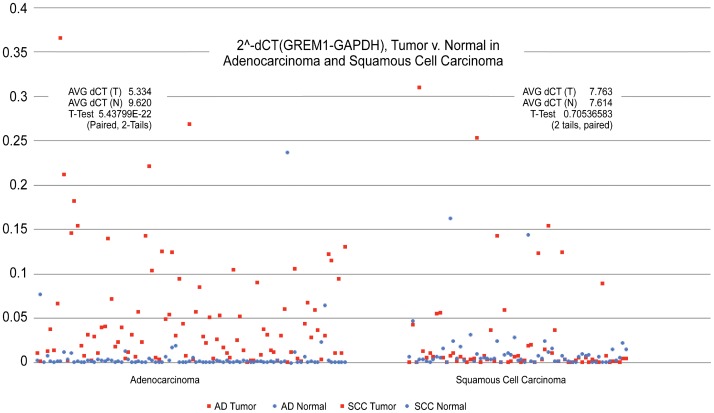
Gremlin is significantly overexpressed in lung AD but not lung SCC. A Taqman qRT-PCR was performed using GREM1 probe and a GAPDH control. Data are represented as GREM1 expression normalized to GAPDH expression for each sample. Gremlin expression was higher in AD specimens than matched normals (N = 96, p = 5.438×10^−22^). There was no significant change in expression in the SCC samples (N = 65, p = 0.705).

To establish the change in expression of Gremlin between tumor and matched normal tissue, a quantitative real-time PCR experiment (Taqman) was performed. Ninety-six adenocarcinoma samples were determined to have significantly higher (p = 1.81E-22) expression of Gremlin when compared to matched normal samples. Sixty-five samples of squamous cell carcinoma were determined to not have any significant difference in expression between tumor and matched normal sample. Change in GAPDH-normalized expression by quantitative real-time PCR is represented in figure 2.

### High Gremlin Protein Expression in lung AD, not SCC

**Figure 3:**
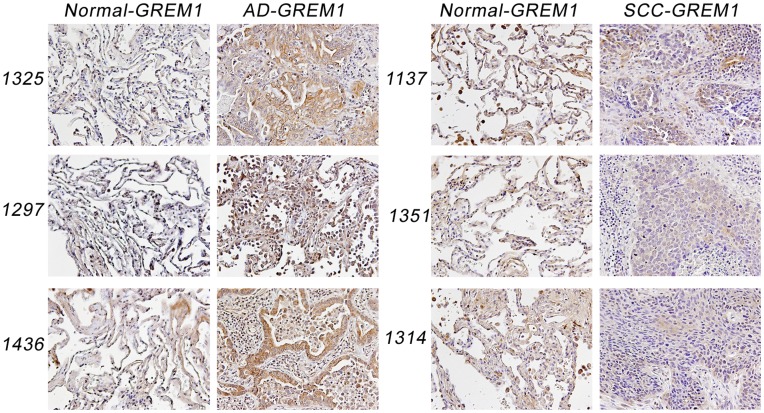
Expression of Gremlin 1 in AD and SCC tumor and normal tissues. AD and SCC tumor samples with matched normal tissue were subjected to immunohistochemical analysis using a rabbit monoclonal anti-human Gremlin antibody. Four representative pairs each of AD and SCC are depicted here. Gremlin appears to be localized in a cytoplasmic dominant manner in AD cancer cells with increased expression in comparison to matched normal tissue. SCC tumor tissue sections do not appear to overexpress Gremlin.

Immunohistochemistry (IHC) was selected to assess if up-regulation of the Gremlin mRNA resulted in a commensurate increase in protein expression. Immunohistochemical staining permitted visualization and localization of Gremlin expression in tumor samples and in matched normals. A total of 24 pairs of adenocarcinoma with matched normal and 8 pairs of squamous cell carcinoma with matched normal tissue were stained for Gremlin protein expression. Gremlin immunoreactivity was observed to be stronger in sections from adenocarcinoma than in matched normal sections. Approximately 67% (16 of 24 samples) of adenocarcinoma samples demonstrated increased Gremlin expression, while 25% (2 of 8 samples) of squamous cell carcinoma samples demonstrated increased expression. Representative immunohistochemical data of adenocarcinoma, squamous cell carcinoma, and matched normal tissue are shown in figure 3.

### Cell Growth and Colony Formation Assay

**Figure 4:**
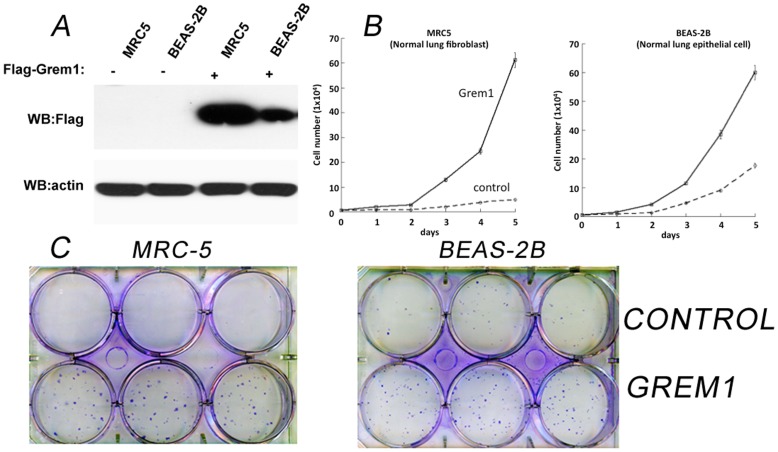
Effect of Grem1 overexpression on MRC5 and BEAS-2B cell proliferation. MRC5 and BEAS-2B pBabepur-flag-Grem1 cells overexpressing flag-tagged Grem1. (A) Grem1 protein expression by immunoblot analysis. B-actin protein expression was the loading control. (B) Growth curve of MRC5 and BEAS-2B cells infected with control vectors or flag-tagged Grem1. Cells (5×10^3^ cells/well) were seeded onto 6-well plates. For each day, three plates were counted and averaged. Error bars show standard deviation of triplicate samples. (two-tailed, unpaired t-test between *GREM1* and vector MRC5 p = 9.88×10^−5^ BEAS-2B p = 9.04×10^−5^) (C) Colony formation assays documenting the effect of Grem1 overexpression on the growth of MRC5 and BEAS-2B cells.

The effects of Gremlin overexpression on cell proliferation were studied using a growth curve assay. The efficiency of Gremlin transfection was checked by immunoblot (Fig. 4a). In the MRC5 cell line, wells containing cells transfected with *GREM1* vector contained an average of 61.2±6.25×10^4^ cells after 5 days while wells containing cells receiving vector alone contained an average of 4.83±0.28×10^4^ cells (two-tailed, unpaired t-test p = 9.88×10^−5^). In the BEAS-2B cell line, wells containing cells transfected with the GREM1 vector contained an average of 50±1.5×10^4^ cells while wells containing cells receiving the control vector contained an average of 17.67±3.18×10^4^ cells after 5 days (two-tailed, unpaired t-test p = 9.04×10^−5^) (Fig. 4b). A colony formation assay was also performed in the MRC-5 and BEAS-2B cell lines. Like the cell growth curve assay, a dramatic increase in colony number was identified in Gremlin-transfected cells (Fig. 4c). To confirm the findings of the colony proliferation assay, the study was again performed using new independent human lung fibroblast (HFL-1) and lung epithelial (NL-20) cell lines. These assays and immunoblot confirmation of transfection are included as figure S1.

### Grem1 Increases Cell Growth via a BMP-2-independent Manner

To assess whether GREM1 overexpression induces proliferation by way of inhibition of BMP-2, we added recombinant BMP-2 to GREM1-transfected MRC-5 cell lines. High concentrations of BMP-2 did not attenuate GREM1 overexpression-induced cell proliferation, indicating that GREM1 increases normal lung cell growth via a BMP-2 independent pathway (Fig. S2).

## Discussion

Lung cancer is the leading cause of cancer death in men and women, both in the U.S. and worldwide, causing deaths – more than breast, prostate, and colorectal cancers combined [28]–[29]. Five-year overall survival remains still less than 20% underscoring the need for a revolution in the management of patients with lung cancer [30]. Although a series of key genes have been reported as playing a role in the tumorigenesis of lung cancer, over 40% of NSCLC patients carry no known mutation or clearly targeted therapeutic indication [5]. And while surgical resection remains a mainstay of therapy, recurrence after surgery remains a serious problem even in stage I patients [31]. We hypothesized that characterization of a new molecular marker with significant overexpression in NSCLC compared to matched normal tissues could identify a novel therapeutic or diagnostic marker. The work reported here describes a potential oncogenic role for Gremlin.

To begin, we conducted a systematic review of published Oncomine data to identify tumor types in which Gremlin may be upregulated in comparison to normal tissue. We identified 24 datasets published to Oncomine that contained both tumor and normal samples suitable for analysis. Comparison of fold-change (t-test, p<.001) between tumor and normal yielded 10 tumor types in which Gremlin was upregulated and 5 in which it was down regulated. Of the datasets in which Gremlin was upregulated, four datasets were of lung tumors (two adenocarcinoma datasets and two squamous cell carcinoma datasets) [9], [10]. Significant upregulation in four lung tumor datasets demonstrated the potential for a significant role of Gremlin in lung cancer.

Following analysis of published microarray data to identify that Gremlin is significantly overexpressed in NSCLC microarray data, we performed quantitative RT-PCR analysis in our 96 AD with matched normal and 65 SCC with matched normal samples. Gremlin expression is significantly increased in lung AD samples compared to matched normal tissues (Fig. 2, p = 1.81E-22). This is consistent with publicly available microarray data that Gremlin is highly overexpressed in tumor [11], [20], [32]. Unlike two published SCC microarray datasets, we did not see a significant up-regulation of SCC compared to matched normal tissue (Fig. 2, p = 0.705) [26], [27]. It is possible that either sample size or a lack of paired samples in published microarray data may contribute to these findings. While our study utilized 65 pairs of SCC and matched normal tissues, samples from Garber et al and Bhattacharjee et al appear to have used 13 and 21 tumor specimens, respectively, but expression levels are compared with normal specimen expression levels pooled from all lung tumor subtypes included in the respective studies [26], [27]. The other possibility is a difference of platform. We conducted a quantitative RT-PCR analysis using a Taqman system while comparative studies utilized a microarray platform.

Next, we checked the protein expression of Gremlin by IHC. We analyzed 24 pairs of AD and 8 pairs of SCC. As shown in Figure 3, Gremlin expression was observed in 67% of the AD samples to be greater than in matched normal slides. In SCC, only 2 of 8 samples demonstrated increased immunoreactivity in tumor specimens (Fig. 3).

We conducted a series of *in vitro* experiments with Gremlin transfection to assess the effect of Gremlin overexpression in lung fibroblast and epithelial cells. Transfection of GREM1 significantly increased cell growth (Fig. 4b) in both normal lung fibroblast (MRC-5) and epithelial (BEAS-2B) cells. Moreover, many colonies were found in GREM1-transfected cells while almost no (MRC-5) or low (BEAS-2B) numbers of colonies were found in vector-transfected cells (Fig. 4c). The use of two additional independent cell lines – HLF-1 and NL-20– reiterated these findings (Fig. S1). These in vitro data support our hypothesis that overexpression of Gremlin in lung cancer is involved in lung tumorigenesis and promotes cell growth and proliferation. While previous work has shown an anti-proliferative role in malignant non-thoracic cell lines, this current study and the recent work of Wang et al suggests an oncogenic role for Gremlin in thoracic malignancies [25]. This difference in tumor-suppressive or tumor-proliferative role for Gremlin appears to be a cell-type dependent effect. Topol et al and Chen et al have shown previously that overexpression of Gremlin can inhibit proliferation in tumor-derived cell lines Daoy (primitive neuroectodermal) and Saos-2 (osteoblastic) [11], [21]. This finding is in concordance with published microarray data showing that Gremlin is consistently downregulated in tumors of the CNS in comparison to matched normals [28]. Thus, overexpression of Gremlin in certain organs such as lung may promote tumor growth, while down-regulation of Gremlin in other organs such as brain may inhibit tumor growth.

The mechanism by which Gremlin enhances proliferation in lung AD remains unclear. Recent developments in the role of BMP2/4 in tumorigenesis and Gremlin’s role in angiogenesis may provide novel directions to elucidate the mechanism by which Gremlin can induce proliferation in lung AD. The ability of Gremlin to antagonize members of the BMP family has previously been well-documented [12]. However, the role of the BMP2/4 proteins in tumorigenesis is controversial [33]. As recently reviewed by Singh and Morris [33], the BMP2/4 proteins are commonly shown to have anti-tumorigenic roles, such as the negative regulation of A549 cell line growth by BMP4 in Buckley, et al [34]. However, BMP2 has been shown in some settings to enhance tumor growth in vivo due to activation of Smad-1/5 [35], [36]. The possibility of a BMP-independent proliferative mechanism for Gremlin is given credence by recent *in vitro* work by Mitola et al and Stabile et al [37], [38]. Mitola et al demonstrate that Gremlin can bind the VEGF receptor-2 (VEGFR2) in a BMP-independent manner. This binding activates VEGFR2 in endothelial cells, culminating in a VEGFR2-dependent angiogenic response *in vitro* and *in vivo*. In addition, Costello et al [39] identified Gremlin as a highly significant member of a 90 gene cohort selectively upregulated in hypoxic primary human pulmonary microvascular cells. We tested whether increased normal lung cell growth induced by GREM1 transfection was BMP2-dependent. MRC-5 fibroblast cells with GREM1 transfection were incubated with BMP2. Addition of high-concentration BMP-2 had no effect on the growth of GREM1-transfected cells (Fig. S2). This suggests that GREM1 increases cell growth through a BMP-independent pathway and further study will be required to better elucidate the mechanism for the tissue-specific function of Gremlin.

In summary, we present evidence that Gremlin is significantly overexpressed in human adenocarcinoma of the lung. Overexpression of Gremlin increases proliferation of both lung epithelial and fibroblast cell lines. Taken together, these studies suggest an oncogenic role for Gremlin in lung adenocarcinoma.

## Materials and Methods

### Ethics Statement

The committee on Human Research (CHR) of UCSF has reviewed and approved the application for the collection of blood, sputum, and tissue samples from patients with suspected or biopsy-proven thoracic malignancies (approval number:10-03352). All samples have been collected under the IRB approval granted by the Committee on Human Research and written informed consents were obtained from all patients in this study.

### Patient Samples

Fresh tumor tissue and adjacent normal tissue samples were obtained at the time of surgical resection of NSCLC patient tumors at the University of California, San Francisco (UCSF) between 1997 and 2007 and deposited in the Thoracic Oncology Tissue Bank. Patients were eligible for the study if they underwent surgical resection of NSCLC at UCSF with curative intent after January 1997. Samples were immediately snap-frozen in liquid nitrogen before storage at −80°C before use. Information on clinical variables and patient follow-up were obtained from a prospectively maintained database as well as review of medical records.

### mRNA Expression – Quantitative Real-time PCR

Total RNA was extracted from snap-frozen samples of lung tumor tissue and matched normal tissue by mechanical homogenization and Trizol. Reverse transcription was performed with random hexamer primers and Superscript II reverse transcriptase (Life Technologies) using 500 ng of total RNA according to the manufacturer’s instructions. One microliter of cDNA was amplified using TaqMan Assays-On-Demand Gremlin gene expression probe (Applied Biosystems, Foster City, CA, assay ID Cf02677902_g1) and GeneAmp 7900 HT Fast sequence detector thermal cycler (Applied Biosystems). Levels of gene expression were determined using the comparative (C_T_) method with samples analyzed in triplicate. Results were then expressed by a modified Livak formula in which GREM1 expression is normalized to the measured expression of the GAPDH gene [40]. Data are represented as 2^-(CT(GREM1)-CT(GAPDH))^ for both tumor and matched normal specimens. The average delta CT of GREM1 in normal samples was 9.620 and an average tumor delta CT was 5.33, suggesting that GREM1 is overexpressed by more than 19 folds in adenocarcinoma.

### Immunohistochemistry

5-micron paraffin-embedded tissue sections from matched normal and tumor tissue samples were deparaffinized in xylene and then rehydrated in graded alcohol. Antigen retrieval was achieved by steaming the tissue sections in citrate buffer for 20 minutes (Biogenex ready-to-use antigen retrieval citra). Slides were then washed in PBS, blocked in goat serum (10% diluted in TBS with 1%BSA) for 1 hour, and then incubated in the primary antibody overnight at 4°C. The primary antibody selected was rabbit monoclonal anti-human Gremlin I (ab90670, ABCam) at a 1 to 500 dilution. Following TBST washes, endogenous peroxidase activity was then quenched with 0.3% hydrogen peroxide in TBS. The Invitrogen ABC-DAB (Histostain Plus Broad Spectrum) kit was then used according to the manufacturer’s protocol. Detection was achieved using a biotinylated anti-goat secondary antibody and DAB chromogen (Invitrogen). The sections were then counterstained with hematoxylin before being mounted with organic media and glass slides. Negative control sections were prepared by the exclusion of the primary antibody to determine the specificity of the staining. Tissue sections were then examined microscopically on an Olympus photomicroscope (Inha, Japan).

### Plasmids

Total RNA was extracted from MRC-5 normal lung fibroblast cells using TRIzol (Invitrogen) and reverse-transcribed using SuperScript One-Step reverse transcription-PCR (RT-PCR). PCR primers were based on human *GREM1* cDNA sequences (Gene ID: 26585) as follows: forward: 5′-CGGAATTCGATGAGCCGCACAGCCTACACGGT-3′ and reverse: 5′-TCGTCTAGATTAATCCAAATCGATGGAATAGCAACGACACTGC-3′) (EcoRI and Xba I linker sequences are underlined). cDNA encoding human Grem1 was subcloned into the EcoRI and XbaI sites of the vectors p3xFlag-CMC-10 (Sigma). The flag-tagged Grem1 cDNA was released by SpeI and XbaI digest and blunt-ended by Mung-Bean nuclease (NEB) before insertion into SnaBI-linearized pBabe.pur retroviral expression plasmid.

### Cells and Generation of Stably Transfected cells

Human MRC-5 lung fibroblast and BEAS-2B lung epithelial cells were obtained from ATCC (www.atcc.org). MRC-5 and BEAS-2B cells expressing flag-tagged Grem1 were generated by transduction with amphotropic retroviruses (pBabe-pur) in the presence of polybrene (8 µg/ml), using high titer retrovirus supernatants derived from Phoenix packaging cells. After infection, MRC-5 and BEAS-2B cells were selected with 2.5 µg/ml puromycin for 6 days and expression of appropriate transgene products confirmed by immunoblotting of cell lysates.

To confirm the findings of the colony formation assays, two additional independent lung fibroblast and epithelial cell lines were studied. Human HFL-1 lung fibroblast and NL-20 lung epithelial cells were obtained from ATCC. HFL-1 and NL-20 cells expressing flag-tagged Grem1 were generated by transduction with amphotropic retroviruses (pBabe-pur) in the presence of polybrene (8 µg/ml), using high titer retrovirus supernatants derived from Phoenix packaging cells. After infection, NL-20 and HFL-1 cells were selected with 0.5 µg/ml puromycin for 6 days and expression of appropriate transgene products confirmed by immunoblotting of cell lysates (Fig. S1).

### Colony Formation Assay

Colony formation assays were performed to investigate the effects of Gremlin overexpression on cell proliferation. Cells (800 cells/well) were seeded onto 6-well plates for 10 days. Culture medium was changed twice a week. After 10 days incubation, the colonies were washed with 1xPBS and fixed with methanol. For the colony formation assay, cells were fixed with methanol and stained with crystal violet (in 25% methanol) for 30 minutes at room temperature.

## Supporting Information

Figure S1 **Effect of Grem1 overexpression on NL-20 and HFL-1 cell proliferation.** (A) Gremlin protein expression by immunoblot analysis. (B) Colony formation assays documenting the effect of Grem1 overexpression on the growth of NL-20 and HFL-1 cells. Cells (1,000/well) were seeded onto 6-well plates and maintained for 10 days.
(TIF)

Figure S2 **Addition of BMP-2 does not affect Grem-1-induced cell growth.** Two different concentration (25 and 75 ng/ul) of a recombinant BMP-2 were tested for their effects on lung fibroblast growth. No significant effect of addition of a recombinant BMP-2 was found, suggesting that Grem1 increases cell growth via a BMP-independent pathway. *No statistical significance (p>0.8, Student’s t-test). Cells were seeded in 6 well plates in triplicates (1×10^4^ cells per plates), incubated with BMP-2, and the cell numbers were counted at the indicated times using a hemocytometer.
(TIF)
