# Supplementary figures and images for: Gremlin is Overexpressed in Lung Adenocarcinoma and Increases Cell Growth and Proliferation in Normal Lung Cells
Source: PLoS One. 2012 Aug 1;7(8):e42264. doi: 10.1371/journal.pone.0042264 (PMC3411619; doi:10.1371/journal.pone.0042264)

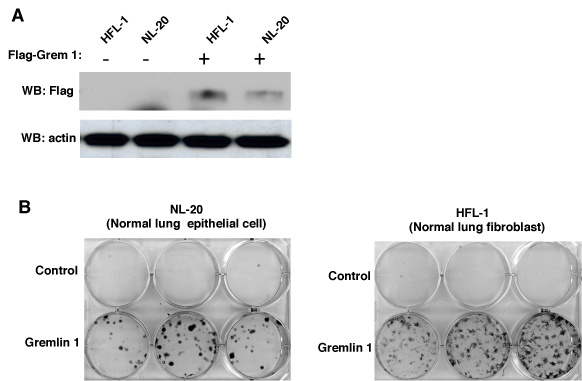

Supplement: Figure S1 — Effect of Grem1 overexpression on NL-20 and HFL-1 cell proliferation. (A) Gremlin protein expression by immunoblot analysis. (B) Colony formation assays documenting the effect of Grem1 overexpression on the growth of NL-20 and HFL-1 cells. Cells (1,000/well) were seeded onto 6-well plates and maintained for 10 days. (TIF) [file pone.0042264.s001.tif]

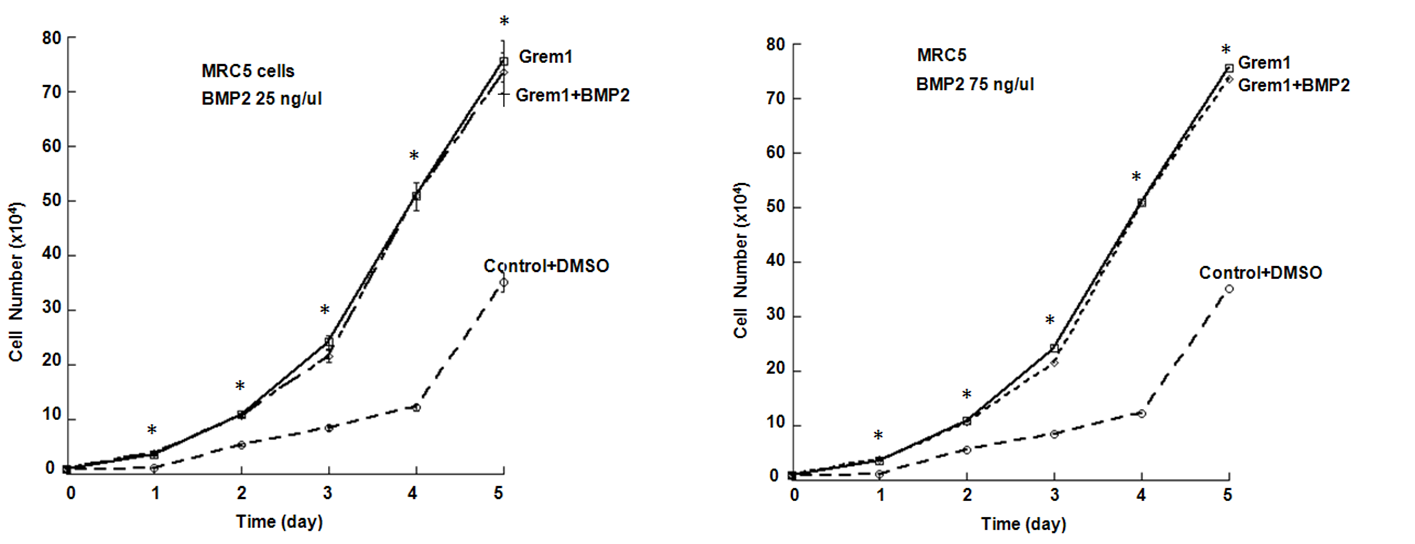

Supplement: Figure S2 — Addition of BMP-2 does not affect Grem-1-induced cell growth. Two different concentration (25 and 75 ng/ul) of a recombinant BMP-2 were tested for their effects on lung fibroblast growth. No significant effect of addition of a recombinant BMP-2 was found, suggesting that Grem1 increases cell growth via a BMP-independent pathway. *No statistical significance (p>0.8, Student’s t-test). Cells were seeded in 6 well plates in triplicates (1×10^4^ cells per plates), incubated with BMP-2, and the cell numbers were counted at the indicated times using a hemocytometer. (TIF) [file pone.0042264.s002.tif]
